# *In vivo* deviation of residual ridge between digital and conventional impressions in the partially edentulous maxilla: a pilot study

**DOI:** 10.7717/peerj.21669

**Published:** 2026-08-24

**Authors:** Shijie Zhang, Shuai Hu, Qing Fang, Jianxiang Tao

**Affiliations:** Shanghai Engineering Research Center of Tooth Restoration and Regeneration & Tongji Research Institute of Stomatology & Department of Prosthodontics, Stomatological Hospital and Dental School, Tongji University, Shanghai, Shanghai, China

**Keywords:** Partially edentulous, Residual ridge, Digital impression, Conventional impressions, Deviation

## Abstract

**Purpose:**

This in vivo study aims to investigate the deviation of the residual ridge between digital and conventional impressions in the partially edentulous maxilla.

**Methods:**

Forty-four patients (22 female, 22 male; 64.8 ± 6.6 years) with partially edentulous maxillae were included in the study. Digital impressions were obtained using an intraoral scanner (TRIOS 3, 3Shape). Conventional impressions were taken with a selective pressure impression technique using the custom tray and a silicone rubber impression material. These impressions were poured with type 4 gypsum. Models were scanned using a desktop scanner (DS-EX Pro C, Shining 3D Dental). The dimensional differences between the digital and conventional impression scans were calculated and defined as the total deviation by the Geomagic Control X software. The vertical displacement at the incisive papilla, mesial, center, and distal points of each residual ridge were measured between the digital and conventional impression scans and defined as the deviations at Point P, Point M, Point C, and Point D, respectively. The residual ridges were divided into distal extension and non-distal extension.

**Results:**

The total deviation and deviation at Point P between the two scans was 0.0 08 mm and 0.0 69 mm, respectively. The total deviation and deviation at Point P exhibit ed no significant difference among patients. For the residual ridge of non-distal extension, the deviations showed no significant difference at Point M, Point C, or Point D (0.032 mm, 0.044 mm, and 0.034 mm, respectively). For the distal extension, the deviations at Point D and Point C (0.136 mm and 0.082 mm, respectively) were significantly greater than that at Point M (0.027 mm) and significantly greater than the deviations at Point D and Point C for non-distal extension (0.034 mm and 0.044 mm, respectively). The deviations of distal extension increased with the number of missing teeth.

**Conclusion:**

The location of the residual ridge had significant effect on its deviation between digital and conventional impressions. This study provides the reference value for the conversion from digital anatomic impressions to digital pressure impressions.

## Introduction

Digital impression technology has been widely adopted into prosthodontic workflows because of its streamlined processes, superior patient comfort, and enhanced operational efficiency (Chochlidakis et al., 2016; Raja et al., 2014; Schmidt, Wöstmann & Schlenz, 2022). Digital impression technology has also been gradually applied in the fabrication of removable partial denture models (Takaichi et al., 2022; Husain et al., 2020; Muralidharan, Schneider & Kotowske, 2024). However, several challenges remain (Bohner et al., 2019; Kim et al., 2017; D’Ambrosio et al., 2023). One such challenge is the accuracy of digital impressions. Conventional impressions place pressure on the residual ridge and can induce morphological changes in the residual ridge (Shin et al., 2019). Conversely, digital impressions never place pressure on the residual ridge because they use a non-contact system (Mangano et al., 2017). However, digital impressions can result in excessive pressure pain on the abutment teeth and food impaction on the denture tissue surface (Hu, Pei & Wen, 2019; Findler et al., 2024).

Previous *in vitro* studies compared the dimensional accuracy of conventional impressions with that of digital impressions and investigated the accuracy of intraoral scanning in different partially edentulous conditions (Güth et al., 2016; Bi et al., 2022; Waldecker et al., 2022). However, these studies did not examine the deviation of the residual ridge between digital and conventional impressions because the *in vitro* partially edentulous model cannot simulate the compressibility of the alveolar mucosa of the residual ridge. By accurately quantifying the morphological deviations at the residual ridge—particularly evaluating the mean deviation of the missing tooth area—between digital and conventional impressions, digital scan data could theoretically be mathematically modified to replicate the functional mucosal morphology achieved *via* conventional pressure impressions. This would represent a potential breakthrough in precise intraoral digital impression techniques. This would be a potential breakthrough in accurate intraoral digital impressions. One *in vivo* study investigated the morphological difference of the residual ridge top under the first molar artificial tooth between digital and conventional impressions in the partially edentulous mandibles of Kennedy Class I and II (Ishioka et al., 2023). However, the stress-bearing region of the maxilla is much larger than that of the mandible. Therefore, the deviation of the residual ridge between digital and conventional impressions in the partially edentulous maxilla might be different from that in the partially edentulous mandible.

Intraoral digital impression technology is based on the superimposition of multiple images (Logozzo et al., 2014; Stefanelli et al., 2021). The anatomical features of the edentulous area, such as the smooth mucosa on the residual ridge, lack distinct and fixed anatomical reference points (Kontis et al., 2021; Rasaie, Abduo & Hashemi, 2021; Leggeri et al., 2023; Fang et al., 2018). Natural teeth located both anterior and posterior to the residual ridge of the non-distal extension could be used as reference points in digital impressions. The residual ridge of the distal extension lacks a posterior reference point, making it more difficult to capture through intraoral scanning.

In this *in vivo* study, digital and conventional impressions were taken on the partially edentulous maxilla. The deviation of the residual ridge between the digital and conventional impressions was examined. The effect of the location of the residual ridge on its deviation and the relationship between the deviation and the number of missing teeth were investigated. The tested null hypothesis was that the location of the residual ridge would have no significant effect on its deviation between digital and conventional impressions.

## Materials and Methods

### Participants

Patients with partially edentulous maxillae who visited the Department of Prosthodontics at the Affiliated Stomatological Hospital of Tongji University between September 2023 and August 2024 were enrolled using a consecutive sampling method. All participants met the criteria for removable partial denture restoration. The inclusion criteria were as follows: patients with an edentulous maxillary ridge for more than three months with a classified class I, class II, class III, or class IV partially edentulous dental arch based on the Kennedy classification of partially edentulous dental arches. The exclusion criteria were as follows: acute dental and periodontal disease; severe mobility of residual teeth; mucosal lesions in the residual ridge; limitation in mouth opening or temporomandibular joint disorders. Forty-four patients (22 female, 22 male) were recruited for this study. The mean participant age was 64.8 ± 6.6 years, as this age range accurately represents the typical clinical population requiring removable partial denture (RPD) rehabilitation. Detailed participant information, including Kennedy classification, gender, age, and number of missing teeth, are exhibited in Table 1. All participants signed informed consent forms and underwent preliminary examinations, including radiographic assessments. This study was approved by the Medical Ethics Committee of the Affiliated Stomatological Hospital of Tongji University (approval number [2023]-SR-38). This study was designed as an *in vivo*, within-subject comparative observational study, where each patient acted as their own control by receiving both digital and conventional impressions. Portions of this text were previously published as part of a preprint (https://doi.org/10.21203/rs.3.rs-6604947/v1).

**Table 1:** Participant demographic data: Kennedy classification, gender, age, and number of missing teeth.

**Kennedy classification**	**Patient’s Gender** ^a^	**Average age**	**Number of missing teeth**	**Number of patients**
Class I	11(M)/8(F)	65.1 ± 6.0	2	1
			4	2
			5	3
			6	2
			7	3
			8	2
			9	2
			11	1
			12	2
			13	1
Class II	6(M)/9(F)	66.5 ± 7.1	2	1
			3	2
			4	2
			5	3
			6	1
			7	2
			9	1
			10	2
			11	1
Class III	2(M)/4(F)	65.6 ± 6.9	3	2
			4	2
			5	1
			8	1
Class IV	3(M)/1(F)	56.5 ± 2.8	2	1
			3	1
			4	1
			5	1

**Notes.**

a M, Male; F, Famale.

### Materials

The following materials were used in this study: intraoral digital impression scanner (TRIOS3, 3Shape, Copenhagen, Denmark); stock tray (SANKIN IMPRESSION TRAY; Dentsply Sirona K.K., Tokyo, Japan); alginate impression material (AROMA FINE PLUS; GC CORPORATION, Tokyo, Japan); silicone rubber impression material (EXAHIFLEX; GC CORPORATION, Tokyo, Japan); type 4 gypsum (Die Stone, Heraeus, Hanau, Germany); extraoral window scanner (DS-EX Pro C, Shining 3D Dental, Hangzhou, China); and surface matching software (Geomagic Control X; 3D Systems, Rock Hill, SC).

### Digital impression

**Figure 1:**
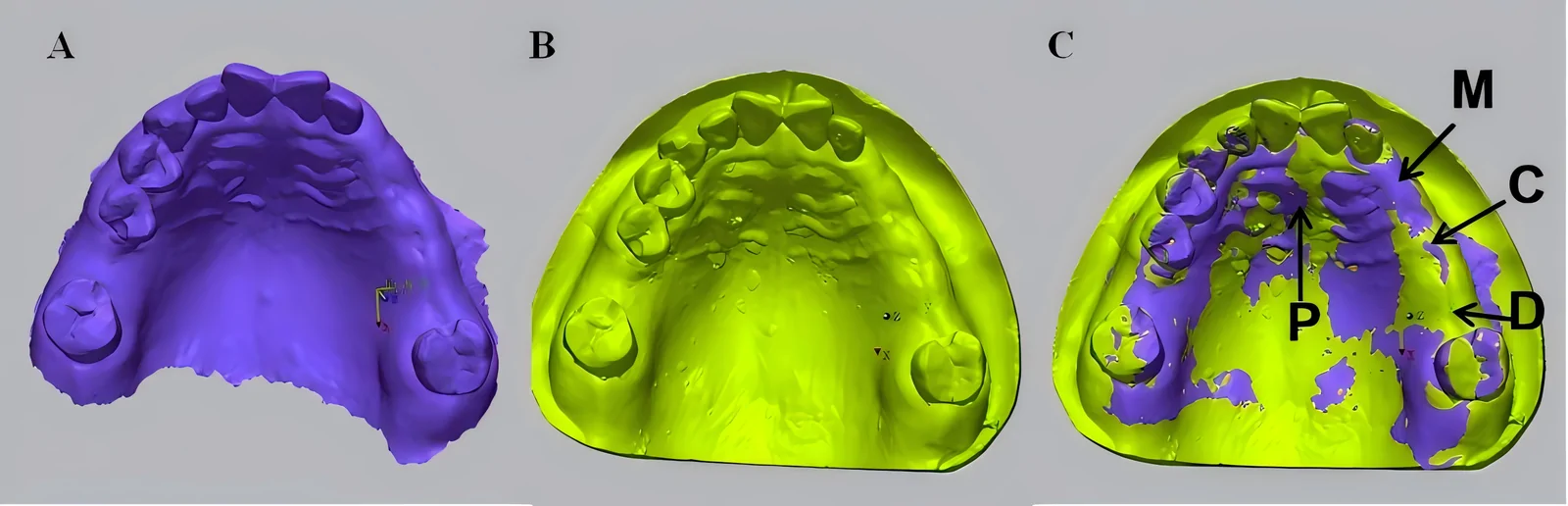
Acquisition and fitting of digital and conventional impression scans. (A) Digital impressions; (B) Conventional pressure impressions; (C) Alignment using the remaining teeth as reference landmarks. Point P: Incisive papilla ; Point M: mesial of residual ridge; Point C: center of residual ridge ; Point D: distal of residual ridge.

To prevent any soft tissue deformation induced by conventional pressure impression trays, digital impressions were performed first for all participants during the same clinical appointment. Before scanning, saliva control and soft tissue management were standardized using cotton rolls and gentle air-drying to ensure optimal scanner conditions. Intraoral image data of maxillary dentition defects were collected from 44 patients by the same prosthodontist using a 3Shape intraoral scanner. There was no recommended scan pattern for the partially edentulous dentition provided by the manufacturer; therefore, the intraoral scans were performed using the protocol demonstrated in a previous study (Çakmak et al., 2022). The scans were initiated from the occlusal surface at the most distal tooth and ridge area on the right side and continued to the occlusal surface at the most distal tooth and ridge area on the left side. This was then followed by a scan of the palatal surface from the left to the right side and then by a scan of the buccal surface from the right to the left side. After the conclusion of the initial scans, any regions with missing data were rescanned until no visible voids were seen on the final scans (Müller et al., 2016). The removable partial denture impressions made using this method are referred to as digital impression scans (Fig. 1A).

### Conventional impression

Immediately following the digital scan, the conventional selective-pressure impression was obtained. A custom tray was fabricated with a stock tray on a diagnostic cast based on a snap impression using a hydrocolloid (alginate) impression material. The definitive impression was taken with a selective pressure impression technique using the custom tray and a silicone rubber impression material. These definitive impressions were poured with type 4 gypsum. The gypsum was mixed under a vacuum and poured under mechanical vibration in accordance with the manufacturer’s instructions. The plaster models were scanned with an extraoral window scanner, and the data were saved in Standard Tessellation Language (STL) format, with the results referred to as conventional impression scans (Fig. 1B). To eliminate inter-operator variability and potential bias, all conventional selective pressure impressions were performed by the same experienced prosthodontist who conducted the intraoral digital scans.

### Accuracy measurements and data analysis

The digital impression scans and the STL files of the conventional impression scans were imported into a surface matching software (Geomagic Control X) and were registered using the remaining natural teeth as stable anatomical reference landmarks. This ensured that any measured deviations precisely represented the true biological displacement of the displaceable soft tissue (residual ridge) rather than alignment algorithm errors. To minimize potential bias, the subsequent 3D data alignment and deviation measurements using the Geomagic Control X software were executed by an independent investigator who was blinded to the specific clinical details of the participants. The overall dimensional differences between the digital and conventional impression scans were calculated as the root mean square (RMS; in mm) to represent the total deviation. Within the software, the mean deviation of the missing tooth area was explicitly quantified to provide a robust assessment of mucosal displacement along the edentulous span. Subsequently, the vertical displacement at the center of the incisive papilla was measured and designated as the deviation at Point P. The vertical displacements at three specific locations on each residual ridge were also measured: the mesial point (Point M, defined as the crest of the residual ridge two mm away from the mesial remaining tooth), the distal point (Point D, defined as the non-distal extension: the crest of the residual ridge two mm away from the remaining distal tooth or distal extension: maxillary tuberosity two mm away from the pterygomaxillary notch), and the center point (Point C, defined as the midpoint between Point M and Point D in the crest of the residual ridge; Fig. 1C). The residual ridges were divided into distal extension and non-distal extension. The definitions of Point M, Point C, and Point D are shown in Table 2.

**Table 2:** Measurement site and sample size.

Sites	Definition	*n*
Point M	The crest of residual ridge 2 mm far from the mesial remaining tooth	69
Point D	Non distal extension: the crest of residual ridge 2mm far from the distal remaining tooth	28
	Distal extension: maxillary tuberosity 2mm far from pterygomaxilay notch	41
Point C	The midpoint between Point M and Point D in the crest of residual ridge	69
Point P	The center of incisive papilla	44

To justify the sample size, an a priori power analysis was conducted using G*Power software (Version 3.1.9.7; University of Düsseldorf, Germany). Based on a pre-experiment involving ten patients focusing on the mean deviation in the residual ridges (0.038 ± 0.086 mm), an estimated effect size of 0.44 was derived. This calculation indicated that a minimum of 42 participants were required to detect a significant difference between digital and conventional impressions with 80% power at an α level of 0.05. A total of 44 patients were included as the sample size of the present study.

Given the within-subject design of this study, deviations between digital and conventional impressions across different measurement sites (Points M, C, and D) were analyzed using a repeated measures analysis of variance (ANOVA), followed by *post-hoc* tests with a Bonferroni correction. Comparisons of deviations between distal and non-distal extension ridges were performed using paired t-tests. Linear regression models were constructed to assess the association between the number of missing teeth and the deviation. Results are reported with regression coefficients (β) and coefficients of determination (R^2^). *P*-values <0.05 were considered statistically significant.

## Results

### Participants and residual ridge

A total of 44 participants (22 female, 22 male; mean age 64.8 ± 6.6 years) were enrolled in this study. The sample size for each type of residual ridge (distal and non-distal) is presented in Table 2.

### The total deviation and deviation at Point P between digital and conventional impression scans

**Figure 2:**
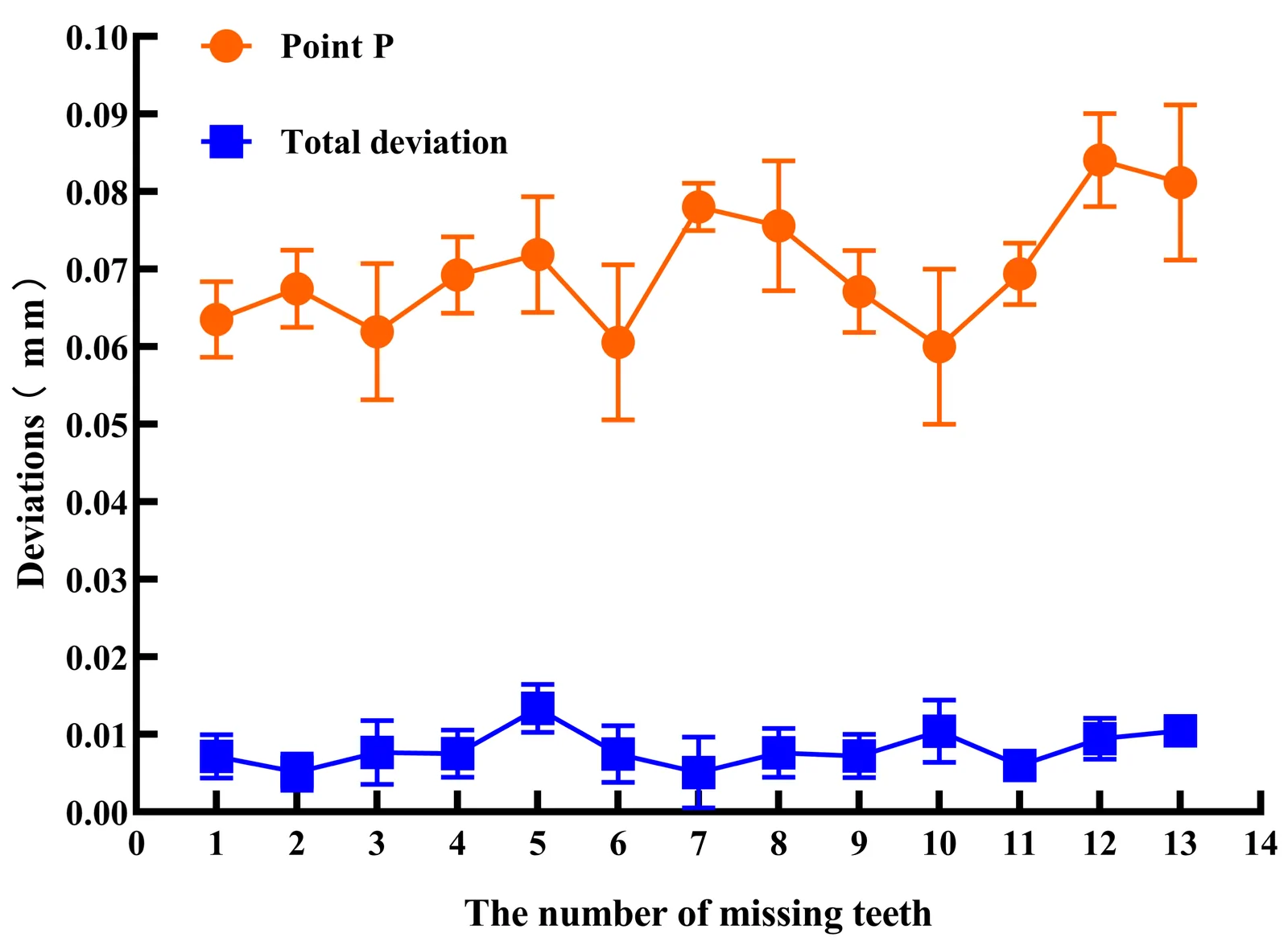
The total deviation and deviation at Point P for different numbers of missing teeth.

The total deviation between the two scans was 0.008 ± 0.003 mm, and the deviation at Point P was 0.069 ± 0.014 mm. Both the total deviation and the deviation at Point P showed no significant difference among participants (*P* > 0.05; Table 3). Correlation analysis found no significant correlation between the deviation and the number of missing teeth for both total deviation and the deviation at Point P (*P* > 0.05; Fig. 2), suggesting that the number of missing teeth did not affect the total deviation or the deviation at Point P.

**Table 3:** Result of total deviation and deviation at Point P between digital and conventional impression scans; X ± SEM.

Scans	Total deviation (mm)	Point P (mm)
Deviation	0.008 ±0.003	0.069 ±0.014
F	1.963	0.716
*P*	0.082	0.727

### The deviation of residual ridges between digital and conventional impression scans

**Figure 3:**
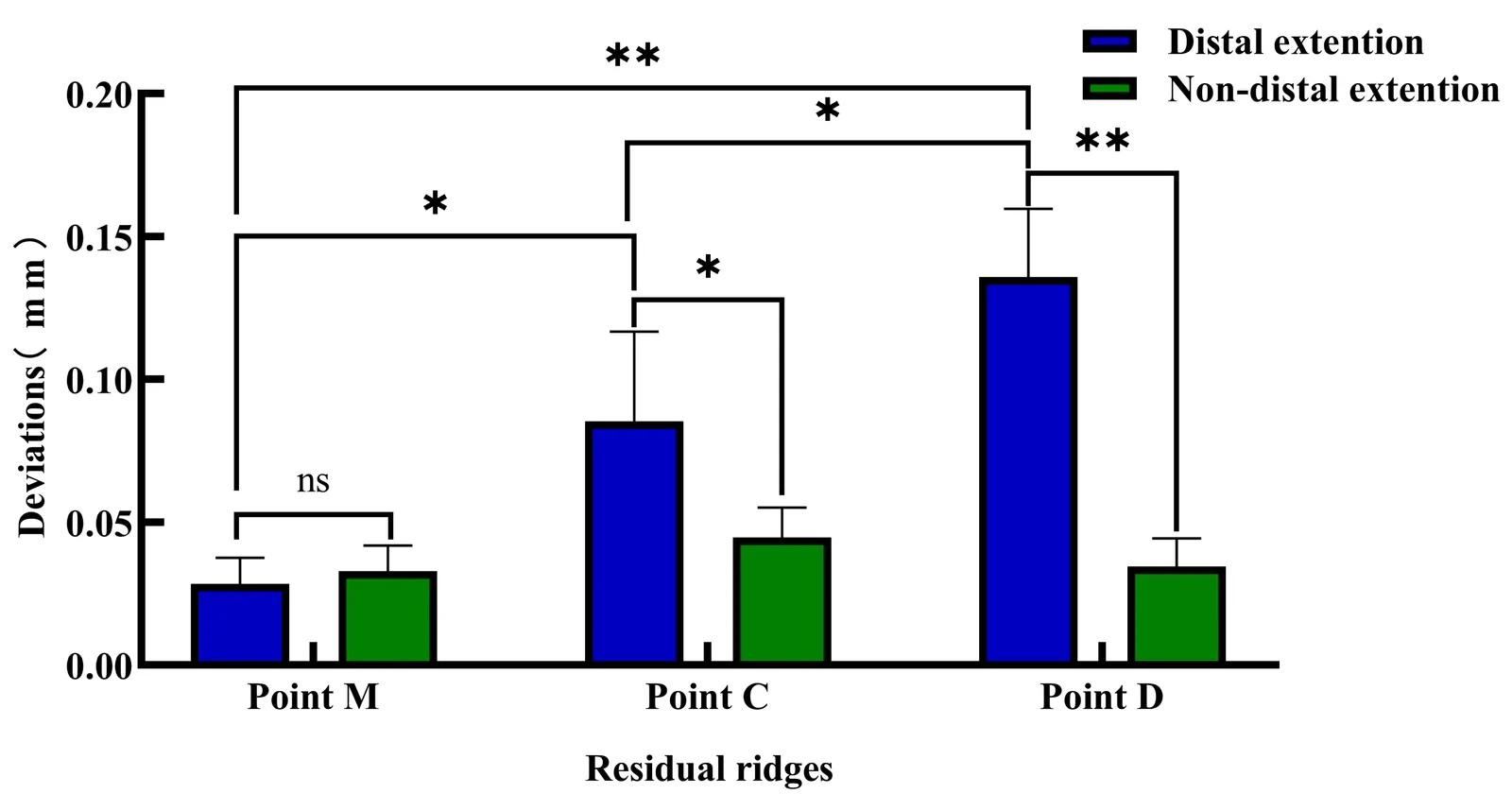
The deviation of residual ridges between digital and conventional impression scans. ns: no significant difference; *: *P* < 0.05; **: *P* < 0.01.

Conventional pressure impressions can induce morphological changes in residual ridges. Therefore, the deviation of residual ridges between digital and conventional impression scans was investigated in detail. For the non-distal extension, there was no significant difference in the deviation of residual ridges among Point M, Point C, and Point D (0.032 mm, 0.044 mm, and 0.034 mm, respectively; Fig. 3). However, for the distal extension, the deviations at Point D and Point C (0.136 mm and 0.082 mm, respectively) were significantly greater than that at Point M (0.027 mm; *P* < 0.05, Fig. 3). The deviation of residual ridges for the distal extension was also compared with that for the non-distal extension. The deviations at Point D and Point C for the distal extension were significantly greater than those for the non-distal extension at Point D and Point C, respectively, which suggests that the location of the residual ridge affected its deviation between the digital and conventional impression scans.

### The relationship between the deviation of residual ridges and the number of missing teeth

**Figure 4:**
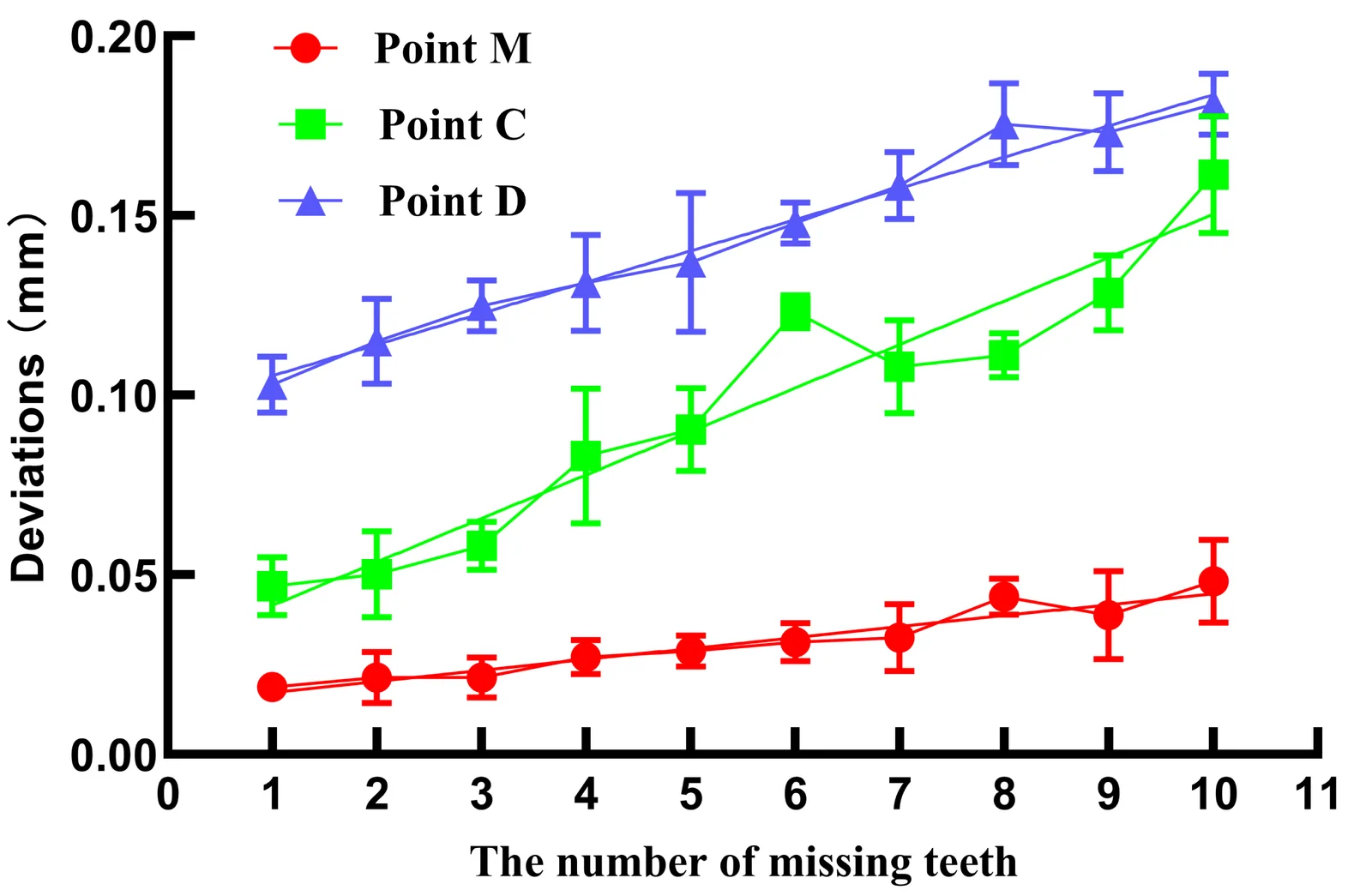
The relationship between the deviation of residual ridges and the number of missing teeth for distal extension.

**Figure 5:**
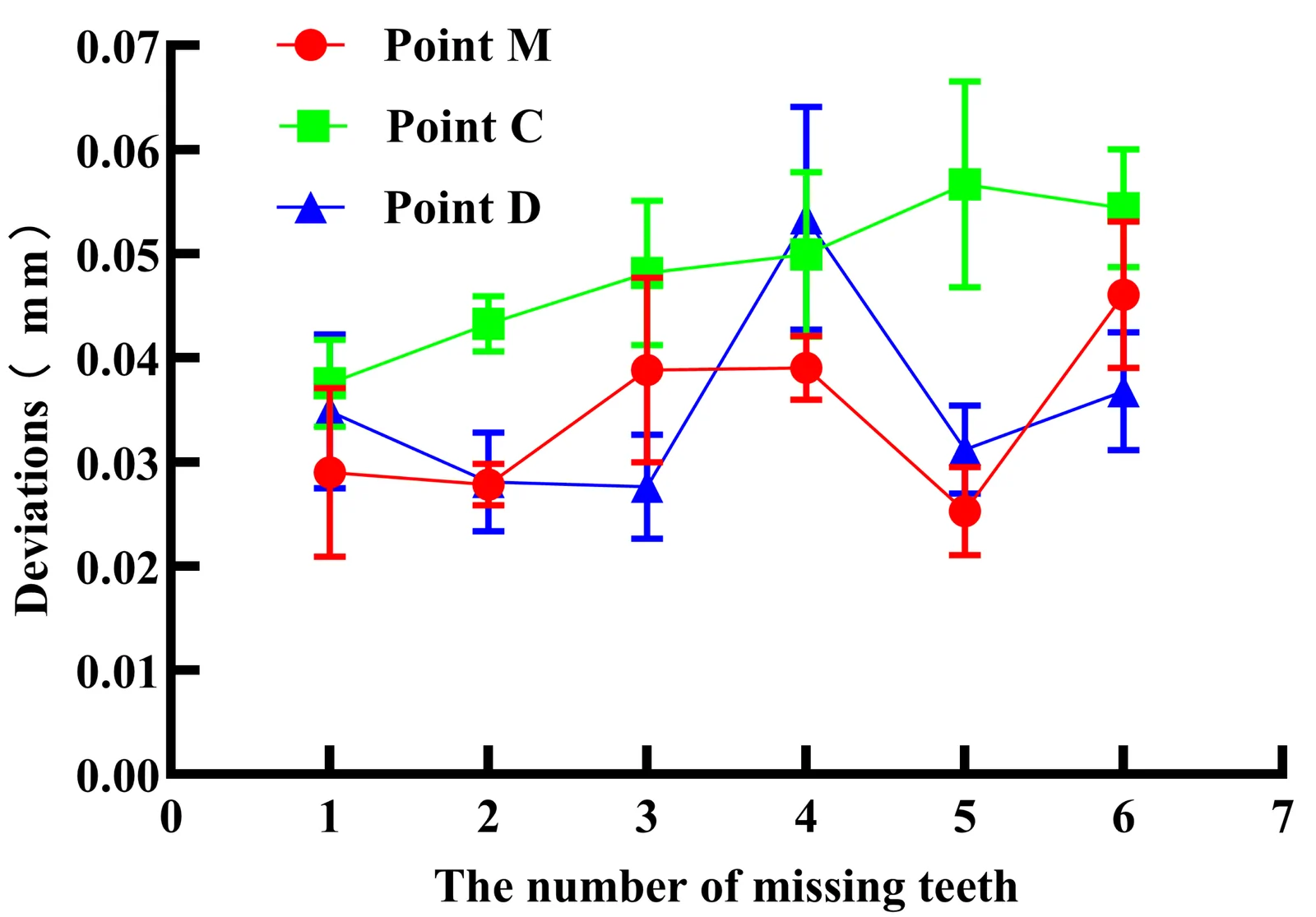
The relationship between the deviation of residual ridges and the number of missing teeth for non-distal extension.

Previous studies indicate that the precision of digital impressions tends to decrease as the edentulous span extends (Lee et al., 2019). Consequently, we investigated the relationship between the deviation of the residual ridges and the number of missing teeth. For the distal extension, evaluating the mean deviation of the missing tooth area through linear regression analysis revealed a significant positive correlation between the length of the edentulous span and the structural deviations at Points M, C, and D (*P* < 0.05, Fig. 4). Specifically, the standardized coefficients (β) and coefficients of determination (R2) progressively increased from the mesial to the distal points (Table 4). The predictive power was moderate for Point M (β = 0.669; mean deviation 95% confidence interval (CI) [0.0240∼0.0300] mm; R2 = 0.447) and Point C (β = 0.686; mean deviation 95% CI [0.0685∼0.0915] mm; R2 = 0.459) and was strongest for the most distal location, Point D (β = 0.719; mean deviation 95% CI [0.1258∼0.1422] mm; R2 = 0.516). This indicates that the deviation at the distal extension increases linearly and significantly with the length of the edentulous span. Conversely, for the non-distal extension, no significant linear relationship was observed between the deviation of residual ridges and the number of missing teeth (*P* > 0.05, Fig. 5).

**Table 4:** Deviations of the distal extension at different points and their linear regression with the number of missing teeth between digital and conventional impression scans; X ± SEM.

Scans	Point M (mm)	Point C (mm)	Point D (mm)
Deviation	0.027 ± 0.01	0.082 ± 0.038	0.136 ± 0.027
β	0.669	0.686	0.719
R^2^	0.447	0.459	0.516
*P*	<0.05	<0.05	<0.05

## Discussion

Currently, the fabrication of removable partial dentures (RPDs) relies on a combination of conventional and digital workflows (Tregerman et al., 2019; Campbell et al., 2017). The rehabilitation of partially edentulous patients typically requires taking conventional pressure impressions, pouring gypsum casts, scanning these models, and performing subsequent computer-aided design and manufacturing (CAD/CAM) based on the digitized data. However, this workflow has not yet achieved full digitalization (Muralidharan, Schneider & Kotowske, 2024; Friel & Waia, 2020; Mai et al., 2022). Conventional pressure impression techniques capture the oral mucosa under appropriate compression to ensure uniform stress distribution across the edentulous mucosa and abutment teeth (Reddy et al., 2013). In contrast, intraoral digital impression technology only captures the mucosal state of the residual ridges without any applied pressure. Furthermore, there is a fundamental difference in how these techniques capture peripheral tissues. Conventional impression materials readily flow into functional vestibules, accurately registering dynamic border extensions. Conversely, intraoral scanners face inherent limitations in capturing mobile vestibular mucosa. The absence of fixed anatomical landmarks in the smooth, edentulous vestibular reflections exacerbates image stitching difficulties and overlapping errors, which significantly contribute to the discrepancies observed in distal extension areas. Additionally, although low-expansion Type 4 dental stone was utilized, the inherent setting expansion and polymerization shrinkage of conventional impression materials must be acknowledged as minor contributing factors to the total recorded deviation. Therefore, the primary objective of this *in vivo* study was to directly compare the deviation of residual ridges between digital and conventional impressions in partially edentulous patients, rather than assessing impression accuracy on rigid in-vitro models as seen in previous research (Gan, Xiong & Jiao, 2016; Mangano et al., 2016; Zarone et al., 2020).

In the present study, the mean total deviation between the digital and conventional impression scans was minimal (0.008 mm), which was substantially smaller than the deviations recorded at Point P and across the residual ridges. This outcome is a direct consequence of the alignment strategy employed; the remaining natural teeth were used as the primary, rigid reference landmarks for best-fit registration. Because natural teeth are non-displaceable, they provide a highly stable coordinate system that yields precise superimposition, thereby effectively minimizing the overall root mean square (RMS) error. This finding underscores that the total deviation across the entire arch does not accurately reflect the localized tissue displacement at specific edentulous sites. Furthermore, the deviation at Point P was greater than that observed in non-distal extensions, yet lower than in distal extensions, suggesting that the incisive papilla undergoes more significant morphological alteration under conventional pressure than the non-distal residual ridges.

Because the anatomical location of the residual ridge significantly influenced its deviation between digital and conventional impression scans, the null hypothesis was rejected. For distal extensions, the deviations at Point D and Point C were significantly greater than those at Point M, and simultaneously exceeded the deviations recorded at corresponding points in non-distal extensions. Furthermore, the deviation in distal extensions increased proportionally with the number of missing teeth. These findings clearly indicate that current digital impressions cannot fully replicate the tissue contours achieved by conventional pressure impressions, particularly in distal extension ridges. This discrepancy is primarily attributed to the compression and subsequent displacement of the mucosa in the distal extension during conventional impression taking (Friel & Waia, 2020; Vahidi, 1978). Additionally, this may be related to intraoral digital impression technology being based on the superimposition of multiple images. The smooth mucosa on the residual ridge lacks distinct and fixed reference points to superimpose multiple images (Kontis et al., 2021; Rasaie, Abduo & Hashemi, 2021; Leggeri et al., 2023; Fang et al., 2018). Unlike non-distal extensions, which are bounded by posterior reference teeth, distal extensions lack posterior landmarks (Schmidt, 2010; Gan, Xiong & Jiao, 2016). Consequently, as the scanning field in a distal extension lengthens, the cumulative deviation induced by frame-overlapping progressively increases (Kontis et al., 2021).

Several strategies have been proposed to enhance the accuracy of digital impressions in edentulous regions. One viable approach is the application of artificial reference markers (Kim et al., 2017). which can be placed in challenging overlap areas, the incisive papilla region, or along the alveolar ridge in extended edentulous spans (Fang et al., 2018; Shimizu et al., 2023). Adopting alternative scanning pathways, such as “Z” or “S” patterns, has also been shown to mitigate image stitching errors (Waldecker et al., 2022; Zarone et al., 2020; Abduo & Elseyoufi, 2018). Additionally, biting occlusal silicone rubber in the edentulous area is often used to simulate functional occlusion, with clinicians quickly completing the mucosal scan once removing the occlusal silicone rubber to achieve more accurate digital pressure impressions (Hirota et al., 2021).

Previous literature has documented tissue displacement resulting from various impression techniques. Vahidi (1978) reported a vertical displacement of approximately 0.200 mm in the distal-extension mucosa of the mandible. In the present study, the deviation at Point D in the maxillary distal extension was 0.136 mm, which is comparatively smaller. This difference may be attributed to the anatomical presence of the hard palate in the maxilla, which provides a larger stress-bearing area and subsequently reduces localized tissue displacement compared to the mandible. More recently, Ishioka et al. (2023) reported a 0.090 mm vertical displacement of the residual ridge beneath the first molar in partially edentulous mandibles; however, their measurement site differed from the more posterior Point D evaluated in our study. From a clinical perspective, an uncompensated gap of 0.136 mm in the distal extension can severely compromise the stability of an RPD. Uncompensated occlusal loading over this discrepancy induces rotational instability pivoting at the rest seat, exerting detrimental torquing forces on the abutment teeth that can accelerate alveolar bone loss and tooth mobility.

As a pilot investigation, these findings establish a preliminary clinical baseline for developing algorithms intended to mathematically convert digital anatomical scans into simulated digital pressure impressions. By continuously aggregating such deviation data into a comprehensive clinical database, future studies may refine computational models to dynamically compensate for mucosal subsidence. If rigorously validated by extensive clinical data, these algorithmic conversions could potentially approximate functional pressure impressions directly from digital anatomic scans, thereby facilitating a fully customized, digital workflow for RPD fabrication. However, such algorithmic conversions remain speculative at this stage and require rigorous future investigation incorporating variables such as mucosal thickness and ridge morphology. This approach facilitates the custom design and fabrication of removable partial dentures.

The present study has several limitations that should be acknowledged. One of the limitations is the small sample size. Only 44 patients with partially edentulous maxillae (22 female, 22 male; 64.8 ± 6.6 years) were recruited in this study. This study also lacks a comparison with a younger, age- and gender-matched edentulous population. Younger patients, possessing thicker and more resilient alveolar ridges, might demonstrate different displacement patterns under conventional pressure impressions, which should be explored in future stratified investigations. Another limitation of the present study is potential confounding variables; specifically, the morphological shape of the residual ridge and the thickness/resiliency of the underlying mucosa were not classified or evaluated in this study. Tissue displacement during conventional impressions is highly sensitive to these anatomical factors. Because further stratification by ridge morphology would have compromised the statistical power of the study’s current sample size, this study focused primarily on the location of the edentulous span. Future investigations should involve larger cohorts stratified by ridge morphology and mucosal thickness and incorporate rigorous reliability testing to establish a more robust clinical database for digital pressure impressions. Another limitation of the present study is the absence of intra-observer or inter-observer reliability testing for the 3D digital measurements. The identification of specific coordinates (such as Points M, C, and D) required manual selection by the operator within the analysis software. Without formal repeatability testing, there remains a potential risk of minor operator bias. This absence means that slight measurement variations could theoretically influence the precise quantitative values of the deviations, particularly in smooth edentulous regions lacking distinct anatomical micro-landmarks. However, given that the 3D superimposition was robustly registered using the rigid and non-displaceable remaining natural teeth as stable reference landmarks, the overall macroscopic trend—specifically, the significantly greater displacement observed in distal extension ridges compared to non-distal extensions—is considered reliable. Future investigations should incorporate rigorous reliability testing to establish a more robust and precise clinical database for digital pressure impressions.

## Conclusions

Within the limitations of this *in vivo* pilot study, the following conclusions can be drawn: The anatomical location of the residual ridge significantly influences the degree of deviation between digital and conventional impressions, with distal extensions exhibiting markedly greater morphological discrepancies than non-distal extensions. The quantitative deviations recorded in this study provide crucial preliminary reference values that may inform future computational algorithms designed to convert digital anatomical impressions into functional digital pressure impressions.

## Supplemental Information

10.7717/peerj.21669/supp-1 Supplemental Information 1 Raw data

## Additional Information and Declarations

### Data Availability

The following information was supplied regarding data availability: The data is available in the Supplemental File.

## References

[ref-1] Abduo J, Elseyoufi M (2018). Accuracy of intraoral scanners: a systematic review of influencing factors. European Journal of Prosthodontics and Restorative Dentistry.

[ref-2] Al-Haj Husain N, Özcan M, Schimmel M, Abou-Ayash S (2020). A digital cast-free clinical workflow for oral rehabilitation with removable partial dentures: a dental technique. Journal of Prosthetic Dentistry.

[ref-3] Bi C, Wang X, Tian F, Qu Z, Zhao J (2022). Comparison of accuracy between digital and conventional implant impressions: two and three dimensional evaluations. The Journal of Advanced Prosthodontics.

[ref-4] Bohner L, Gamba DD, Hanisch M, Marcio BS, Neto PT, Laganá DC, Sesma N (2019). Accuracy of digital technologies for the scanning of facial, skeletal, and intraoral tissues: a systematic review. Journal of Prosthetic Dentistry.

[ref-5] Campbell SD, Cooper L, Craddock H, Hyde TP, Nattress B, Pavitt SH, Seymour DW (2017). Removable partial dentures: the clinical need for innovation. Journal of Prosthetic Dentistry.

[ref-6] Çakmak G, Yilmaz H, Treviño Santos A, Kökat AM, Yilmaz B (2022). Effect of scanner type and scan body location on the accuracy of mandibular complete-arch digital implant scans: an *in vitro* study. Journal of Prosthodontics.

[ref-7] Chochlidakis KM, Papaspyridakos P, Geminiani A, Chen C-J, Feng IJ, Ercoli C (2016). Digital *versus* conventional impressions for fixed prosthodontics: a systematic review and meta-analysis. Journal of Prosthetic Dentistry.

[ref-8] D’Ambrosio F, Giordano F, Sangiovanni G, Di Palo MP, Amato M (2023). Conventional *versus* digital dental impression techniques: what is the future? An umbrella review. Prosthesis.

[ref-9] Fang J-H, An X, Jeong S-M, Choi B-H (2018). Digital intraoral scanning technique for edentulous jaws. Journal of Prosthetic Dentistry.

[ref-10] Findler M, Perzon O, Almoznino G, Zini A, Sharav Y, Czerninski R, Aframian D, Haviv Y (2024). Unveiling denture-induced oral lesions: a comprehensive study on classification and pain assessment. Journal of Oral Rehabilitation.

[ref-11] Friel T, Waia S (2020). Removable partial dentures for older adults. Primary Dental Journal.

[ref-12] Gan N, Xiong Y, Jiao T (2016). Accuracy of intraoral digital impressions for whole upper jaws, including full dentitions and palatal soft tissues. PLOS ONE.

[ref-13] Güth J-F, Edelhoff D, Schweiger J, Keul C (2016). A new method for the evaluation of the accuracy of full-arch digital impressions *in vitro*. Clinical Oral Investigations.

[ref-14] Hirota Y, Tawada Y, Komatsu S, Watanabe F (2021). Effect of impression holding time and tray removal speed on the dimensional accuracy of impressions for artificial abutment tooth inclined. Odontology.

[ref-15] Hu F, Pei Z, Wen Y (2019). Using intraoral scanning technology for three-dimensional printing of kennedy class i removable partial denture metal framework: a clinical report. Journal of Prosthodontics.

[ref-16] Ishioka Y, Wada J, Kim EY, Sakamoto K, Arai Y, Murakami N, Yamazaki T, Takakusaki K, Hayama H, Utsumi M, Inukai S, Wakabayashi N (2023). Morphological comparison of residual ridge in impression for removable partial denture between digital and conventional techniques: a preliminary *in-vivo* study. Journal of Clinical Medicine.

[ref-17] Kim J-E, Amelya A, Shin Y, Shim J-S (2017). Accuracy of intraoral digital impressions using an artificial landmark. Journal of Prosthetic Dentistry.

[ref-18] Kontis P, Güth J-F, Schubert O, Keul C (2021). Accuracy of intraoral scans of edentulous jaws with different generations of intraoral scanners compared to laboratory scans. The Journal of Advanced Prosthodontics.

[ref-19] Lee J-H, Yun J-H, Han J-S, Yeo I-SL, Yoon H-I (2019). Repeatability of intraoral scanners for complete arch scan of partially edentulous dentitions: an *in vitro* study. Journal of Clinical Medicine.

[ref-20] Leggeri A, Carosi P, Mazzetti V, Arcuri C, Lorenzi C (2023). Techniques to improve the accuracy of intraoral digital impression in complete edentulous arches: a narrative review. Applied Sciences.

[ref-21] Logozzo S, Zanetti EM, Franceschini G, Kilpelä A, Mäkynen A (2014). Recent advances in dental optics—Part I: 3D intraoral scanners for restorative dentistry. Optics and Lasers in Engineering.

[ref-22] Mai HY, Mai H-N, Kim H-J, Lee J, Lee D-H (2022). Accuracy of removable partial denture metal frameworks fabricated by computer-aided design/ computer-aided manufacturing method: a systematic review and meta-analysis. Journal of Evidence-Based Dental Practice.

[ref-23] Mangano F, Gandolfi A, Luongo G, Logozzo S (2017). Intraoral scanners in dentistry: a review of the current literature. BMC Oral Health.

[ref-24] Mangano FG, Veronesi G, Hauschild U, Mijiritsky E, Mangano C (2016). Trueness and precision of four intraoral scanners in oral implantology: a comparative *in vitro* study. PLOS ONE.

[ref-25] Müller P, Ender A, Joda T, Katsoulis J (2016). Impact of digital intraoral scan strategies on the impression accuracy using the TRIOS Pod scanner. Quintessence International.

[ref-26] Muralidharan C, Schneider RL, Kotowske S (2024). CAD-CAM denture teeth made on cast metal removable partial denture frameworks. Journal of Prosthetic Dentistry.

[ref-27] Raja SR, Dutta A, Jain SK, Dewan H, Thomas V, Jose AT (2014). *In vitro* comparative analysis of digital versus conventional impressions in fixed prosthodontics. Journal of Pharmacy and Bioallied Sciences.

[ref-28] Rasaie V, Abduo J, Hashemi S (2021). Accuracy of intraoral scanners for recording the denture bearing areas: a systematic review. Journal of Prosthodontics.

[ref-29] Reddy SM, Mohan CA, Vijitha D, Balasubramanian R, Satish A, Kumar M (2013). Pressure produced on the residual maxillary alveolar ridge by different impression materials and tray design: an *in vivo* study. The Journal of Indian Prosthodontic Society.

[ref-30] Schmidt A, Wöstmann B, Schlenz MA (2022). Accuracy of digital implant impressions in clinical studies: a systematic review. Clinical Oral Implants Research.

[ref-31] Schmidt V (2010). 3D dental camera for recording surface structures of a measuring object by means of triangulation. International Publication WO Patent.

[ref-32] Shimizu T, Tasaka A, Wadachi J, Yamashita S (2023). A new proposal for improving the accuracy of intraoral scanning for partially edentulous residual ridge. Journal of Prosthodontic Research.

[ref-33] Shin JO, Ko KH, Huh YH, Cho LR, Park CJ (2019). Displacement of simulated flabby tissue by different tray designs and impression materials. Journal of Prosthodontics.

[ref-34] Stefanelli LV, Franchina A, Pranno A, Pellegrino G, Ferri A, Pranno N, Di Carlo S, De Angelis F (2021). Use of intraoral scanners for full dental arches: could different strategies or overlapping software affect accuracy?. International Journal of Environmental Research and Public Health.

[ref-35] Takaichi A, Fueki K, Murakami N, Ueno T, Inamochi Y, Wada J, Arai Y, Wakabayashi N (2022). A systematic review of digital removable partial dentures. Part II: CAD/CAM framework, artificial teeth, and denture base. Journal of Prosthodontic Research.

[ref-36] Tregerman I, Renne W, Kelly A, Wilson D (2019). Evaluation of removable partial denture frameworks fabricated using 3 different techniques. Journal of Prosthetic Dentistry.

[ref-37] Vahidi F (1978). Vertical displacement of distal-extension ridges by different impression techniques. Journal of Prosthetic Dentistry.

[ref-38] Waldecker M, Rues S, Behnisch R, Rammelsberg P, Bömicke W (2022). Effect of scan-path length on the scanning accuracy of completely dentate and partially edentulous maxillae. Journal of Prosthetic Dentistry.

[ref-39] Zarone F, Ruggiero G, Ferrari M, Mangano F, Joda T, Sorrentino R (2020). Comparison of different intraoral scanning techniques on the completely edentulous maxilla: an *in vitro* 3-dimensional comparative analysis. Journal of Prosthetic Dentistry.

